# Stereological Analysis of Early Gene Expression Using Egr-1 Immunolabeling After Spreading Depression in the Rat Somatosensory Cortex

**DOI:** 10.3389/fnins.2019.01020

**Published:** 2019-09-25

**Authors:** Marcia Consentino Kronka Sosthenes, Daniel Guerreiro Diniz, Jay Roodselaar, Ricardo Abadie-Guedes, Fabíola de Carvalho Chaves de Siqueira Mendes, Taiany Nogueira Fernandes, Jackson Cioni Bittencourt, Cristovam Wanderley Picanço Diniz, Daniel Clive Anthony, Rubem Carlos Araújo Guedes

**Affiliations:** ^1^Laboratório de Investigações em Neurodegeneração e Infecção, Instituto de Ciências Biológicas, Hospital Universitário João de Barros Barreto, Universidade Federal do Pará, Belém, Brazil; ^2^Laboratory of Experimental Neuropathology, Department of Pharmacology, University of Oxford, Oxford, United Kingdom; ^3^Laboratório de Neuroanatomia Química, Departamento de Anatomia, Universidade de São Paulo, São Paulo, Brazil; ^4^Laboratório de Fisiologia da Nutrição Naíde Teodósio, Departamento de Nutrição, Universidade Federal de Pernambuco, Recife, Brazil; ^5^Curso de Medicina, Centro Universitário do Estado do Pará, Belém, Brazil; ^6^Núcleo de Neurociências e Comportamento, Instituto de Psicologia, Universidade de São Paulo, São Paulo, Brazil

**Keywords:** cortical spreading depression, somatosensorial cortex, Egr-1, optical fractionator, contralateral hemisphere

## Abstract

Early growth response-1 (Egr-1), defined as a zinc finger transcription factor, is an upstream master switch of the inflammatory response, and its expression can be used to investigate the spatial and temporal extent of inflammatory changes in the brain. Cortical spreading depression (CSD) is characterized as a slowly propagating (2–5 mm/min) depolarization wave through neurons and astrocytes in humans that contributes to migraines and possibly to other brain pathologies. In rodents, CSD can be induced experimentally, which involves unilateral depolarization that is associated with microglial and astrocyte responses. The impact of CSD on structures beyond the affected hemisphere has not been explored. Here, we used an optical fractionator method to investigate potential correlations between the number of and period of the eletrophysiologic record of CSD phenomena and Egr-1 expression in ipsilateral and contralateral hemispheres. CSD was elicited by the restricted application of a 2% KCl solution over the left premotor cortex. Electrophysiological events were recorded using a pair of Ag/AgCl agar-Ringer electrodes for 2 or 6 h. An optical fractionator was applied to count the Egr-1 positive cells. We found that CSD increased Egr-1 expression in a time- and event-dependent manner in the ipsilateral/left hemisphere. Although CSD did not cross the midline, multiple CSD inductions were associated with an increased number of Egr-1 positive cells in the contralateral/right hemisphere. Thus, repeated CSD waves may have far reaching effects that are more global than previously considered possible. The mechanism of contralateral expression is unknown, but we speculate that callosal projections from the depolarized hemisphere may be related to this phenomenon.

## Introduction

The Egr-1 gene is a kind of immediate early genes (IEGs) that can be stimulated by a range of extracellular signaling molecules, including growth factors, neurotransmitters, hormones, cytotoxic metabolites and differentiation factors (Lim et al., 1987; Milbrandt, 1987; Thiel and Cibelli, 2002). Egr-1 is widely expressed and regulates a variety of cellular processes, such as cell proliferation and growth (Thiel and Cibelli, 2002), and neuronal differentiation and inflammation (Peng et al., 2017; Yu et al., 2018). Egr-1 activation can be mediated by several factors, but the downstream consequences of its activation have yet to be elucidated. Recently, Egr-1 was suggested to play a role in apoptosis (Thiel and Cibelli, 2002). The high-mobility group box 1 (HMGB1) protein, which is a ubiquitously expressed non-histone DNA binding nuclear protein, has been related as an inflammatory mediator in CSD and migraine (Karatas et al., 2013; Takizawa et al., 2016).

The Egr-1 gene is thought to couple extracellular signals to long-term cellular responses by altering the expression of Egr-1 target genes (Thiel and Cibelli, 2002), when the products of this may then lead to physiological alterations. Mo et al. (2015) suggested that neuronal activity-dependent upregulation of Egr-1 might alter the subtypes of GABA-A receptors, which are involved to the maintenance of the homeostatic excitatory and inhibitory balance for synaptic strength regulation. It was further suggested that Egr-1 upregulation correlates with changes in dendritic spine density. In this scenario, Egr-1 may regulate the expression of drebrin, an actin-binding protein that is highly expressed in dendritic spines (Cho et al., 2017).

Cortical spreading depression (CSD) immediately upregulates Egr-1 in the brain (McKee et al., 2006). However, little is known about Egr-1 expression over an extended time frame. Indeed, the potential mechanisms linking transient electrical changes and long-lasting structural reorganization remain largely unexplored (Sommerlandt et al., 2018). Here we investigated the relationship between electric transitory events and structural reorganization by inducing CSD and monitoring brain electrical activity for 2 and 6 h after induction to identify potential correlations with Egr-1 expression. The effects on neural cells *in vivo* remain limited.

Cortical spreading depression was first described in rabbits by Leão in 1944 (Dussor, 2015) and is defined as a slowly propagating wave (2–5 mm/min) of almost-complete neuronal and glial cell depolarization followed by suppress electrical activity of neurons from cortex (Pietrobon and Moskowitz, 2014; Takizawa et al., 2016). Due to the massive and synchronous depolarization of neurons and glia that occur during CSD, some authors have opted for the term spreading depolarization, highlighting that the depression of cellular activity that follows the CSD wave is also important (Leao, 1947; Kadam and Dudek, 2016). CSD can be observed in damaged brain regions and is related to neurological disorders, such as migraine aura (Fabricius et al., 2006; Hosseini-Zare et al., 2017), brain trauma (Lauritzen et al., 2011; Hosseini-Zare et al., 2017) and epileptic seizures, but it can also be induced experimentally using chemical stimulation (Guedes et al., 2013, 2017; Torrente et al., 2014).

In the present work, we investigated the expression of immediate early response genes in the somatosensory cortex following CSD induction. We estimated Egr-1 immunolabeling density using stereological analysis at the infragranular layers of the parietal cortex in both contralateral and ipsilateral hemispheres. We examined Egr-1 expression to circumvent potential KCl-induced damage on the supragranular layers and used of somatosensory cortex to record CSD, since this is the first area to be invaded by the propagating wave that is induced in the frontal cortex. Although CSD acutely increases early gene expression in the brain, there have been no detailed quantitative analyses that used unbiased stereological methods to investigate this further. We tested the hypothesis that there is a greater number of Egr-1–positive cells when there are more CSD episodes. Toward this end, we induced either a lower or a higher number of CSD episodes and then estimated the density of Egr-1-immunolabeled cells in the contralateral and ipsilateral hemispheres. We also investigated whether this expression was maintained over the duration of electrophysiological recording.

## Materials and Methods

This study used 39 male Wistar rats that were 90 to 120 days old. The rats were treated in accordance with the regulations of the Ethics Committee for Animal Research of the Federal University of Pernambuco, Brazil (protocol approval no. 0037/2016), where the CSD experiments were conducted. All experimental procedures were in accordance with the “Principles of Laboratory Animal Care” (NIH; Bethesda, MD, United States). The animals were housed in standard polypropylene cages (51 × 35.5 × 18.5 cm) in a temperature-controlled room (22 ± 1°C) under a 12:12 light:dark cycle. The animals had free access to water and to a commercial laboratory chow diet.

There were 6 groups of animals based on two recording times (2 and 6 h) and three CSD exposure levels according to the number of CSD episodes: zero (sham/no exposure), low number of episodes (low intensity exposure), and high number of episodes (high intensity exposure). We analyzed the right (CSD-free or contralateral side) and the left (CSD or ipsilateral side) hemispheres separately; thus, for our analysis, there were 12 datasets of data from the 6 groups of rats (Table 1).

**TABLE 1:** Experimental animal groups in which the contralateral (*n* = 6) and the ipsilateral (*n* = 6) hemispheres were analyzed with two recording durations (2 and 6 h) and three levels of cortical spreading depression (CSD) episodes.

	**2 h recording**	**6 h recording**
Low number of CSD episodes	Right hemisphere (RH)	Right hemisphere (RH)
(2 to 5 episodes)	Left hemisphere (LH)	Left hemisphere (LH)
High number of CSD episodes	Right hemisphere (RH)	Right hemisphere (RH)
(6 to 11 episodes)	Left hemisphere (LH)	Left hemisphere (LH)
Sham	Right hemisphere (RH)	Right hemisphere (RH)
	Left hemisphere (LH)	Left hemisphere (LH)

*The CSD exposure levels were zero episodes (sham), a low number of episodes (2–5 episodes), and a high number of episodes (6–11 episodes).*

**FIGURE 1:**
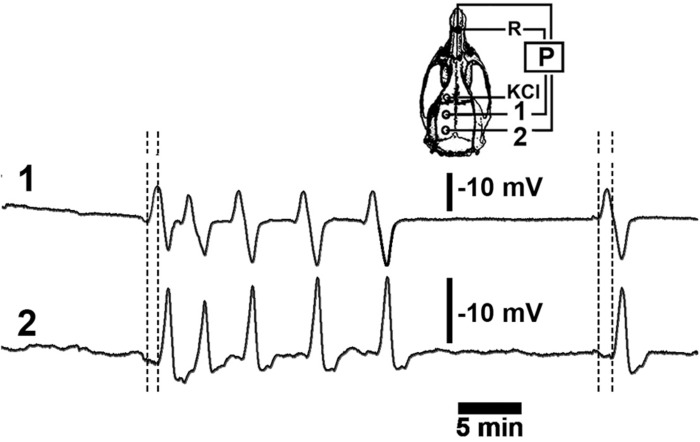
A representative recording of DC slow potential changes during cortical spreading depression (CSD). Six episodes of CSD were elicited by applying a cotton ball soaked with a 2% KCl solution on the frontal cortex over a 30-min period at the point marked “KCl” on the skull diagram. The left hemisphere (skull diagram) shows recording positions 1 and 2 on the parietal cortex as well as the position of the reference electrode (R) on the nasal bones. Vertical calibration bars equal to 10 mV for the P recordings. Horizontal bar equals 5-min.

The CSD recording sessions were conducted under i.p. anesthesia using a mixture of 1 g/kg urethane plus 40 mg/kg chloralose (Sigma) as previously described (Abadie-Guedes et al., 2008; Guedes et al., 2013, 2017). Three trephine holes were made on the ipsilateral side of the skull, parallel to the midline. The first hole was localized on the frontal diploe (approximately 2 mm in diameter), and this was where we applied the stimulus (2% KCl solution) to elicit CSD; the 2 other holes were made in the parietal squama (3 to 4 mm in diameter) where recording electrodes were placed (As stereotaxic coordinates, it was applied: stimulation hole with KCl, AP = +1; ML = 1; recording position 1: AP = - 2; ML = 1.5; recording position 2: AP = - 6; ML = 2) (Figure 1). The centers of the holes were separated by about 5 to 7 mm. The rectal temperature was continuously monitored and maintained at 37 ± 1°C using a heating blanket. A cotton ball (approximately 2-mm in diameter) soaked with a 2% KCl solution (270 mM) was applied intermittently to obtain a low or high number of CSD episodes. The cotton ball was used for 30 min, and recording continued for up to 2 or 6 h (for the short and long recording groups, respectively). The changes in slow direct current potential (DC potential) and the decrease in spontaneous cortical electrical activity associated with CSD were continuously recorded at the 2 parietal holes using a pair of Ag/AgCl agar-Ringer electrodes, as described previously (Abadie-Guedes et al., 2008; Guedes et al., 2013; Mo et al., 2015) (Figure 1). Both holes for the application of KCl and the placement of the 2 electrodes were made on the intact dura mater.

The changes in DC potential were recorded by connecting the electrodes to DC amplifiers (DA100C, General Purpose Transducer amplifier, BIOPAC System, United Kingdom), and the EEG (electroencephalogram) was recorded using AC amplification (EEG 100C amplifier, BIOPAC System, United Kingdom; band pass filters were set to the 1 to 35 Hz range). Both the DC recording and the EEG were computer-digitized (MP150 System with AcqKnowledge software, version 4.1). The sham groups only received anesthesia, and the recording was performed by the same way; however, the 2% KCl solution was not applied.

### Perfusion and Histology

As previously described (Carvalho-Paulo et al., 2017), using deep anesthesia, all recorded animals were perfused by a cardiac via with a 0.9% saline solution followed by aldehyde fixative (4% formaldehyde in 0.1 M phosphate buffer, pH 7.2–7.4). The brains were carefully dissected, and the ipsilateral and contralateral sides were separated, post-fixed in 4% formaldehyde in phosphate buffer, and sliced using a Vibratome (Leica VT1000M) in the sagittal plane. The anatomical series of 100-μm thick sections were free-floating immunolabeled with anti-Egr-1 antibody (SC-515830, Santa Cruz Biotechnology Inc.) and the sections were organized on glass slides pre-prepared with a mixture of aqueous solution of gelatin (10%) and chromium potassium sulfate (0.5%). Following, the sections were air-dried at room temperature, dehydrated, and cleared using an alcohol and xylene series. It is important to say that although the thickness used for vibration cutting was 100-μm, the optical fractionator methodology employed (see below) allowed us to estimate the number of Egr-1 positive cells working at a thickness of approximately 30-μm, even with exclusion from the guard zone.

This ensured that surface imperfections associated with the processing of material did not interfere in the identification of objects of interest, and in the transit between top and bottom of the section the penetration of antibody related to immunohistochemistry was confirmed (Bento-Torres et al., 2017).

### Immunohistochemistry

The protocols applied by the present study were previously used by Carvalho-Paulo et al. (2017), with some adaptations related to antibody. Free-floating sections were treated in 0.2 M boric acid (pH 9.0) for antigenic retrieval by incubation in at 70°C for 60 min. The sections were then washed in PBS-Triton (PBST; 0.1% Triton); washed 3 times × 2 min each in PBS; immersed for 1 h in 10% casein; washed 3 times × 2 min each in PBS; and incubated for 72 h at 4°C with the anti-Egr-1 antibody (SC-515830, Santa Cruz Biotechnology Inc.) diluted 1:500 in PBST (0.3% Triton) with gentle and continuous agitation. “After washing in PBST (0.1% Triton), the sections were incubated overnight in rabbit anti-rat secondary antibody solution (Vector Laboratories, Burlingame, CA, United States) diluted 1:250 in PBST (0.3% Triton). They were then incubated in 0.3% hydrogen peroxide for 15 min; washed 3 times × 2 min each in PBST; and incubated for 60 min in avidin–biotin–peroxidase complex solution [Vector Laboratories, Burlingame, CA, United States; 37.5 μl A + 37.5 μl B in 13.12 ml PBST (0.3% Triton)]. After a 2-min wash in PBS, the glucose-oxidase-DAB-nickel method (Shu et al., 1988) was used to visualize Egr-1–immunolabeled cells. The reaction was interrupted after cells were detected under the microscope. Sections were rinsed 3 × 5 min each in 0.1 M PBS, mounted on gelatinized slides, dehydrated in an alcohol and xylene series, and mounted on a coverslip with DPX mounting medium (Sigma-Aldrich) (Carvalho-Paulo et al., 2017). Five animals from each experimental group and three animals from the sham groups were used for the stereological analysis of Egr-1 immunohistochemistry.

Sections from 2 h high number of CSD episodes (ipsilateral and contralateral hemispheres), 6 h high number of CSD episodes (ipsilateral and contralateral hemispheres) and 2 h low number of CSD episodes (ipsilateral and contralateral hemispheres) were immunolabeled for anti-Iba-1, using the same solutions and protocol to guarantee comparability between them, to illustrate microglia morphological differences between right and left hemispheres.

### Stereology and Statistics

**FIGURE 2:**
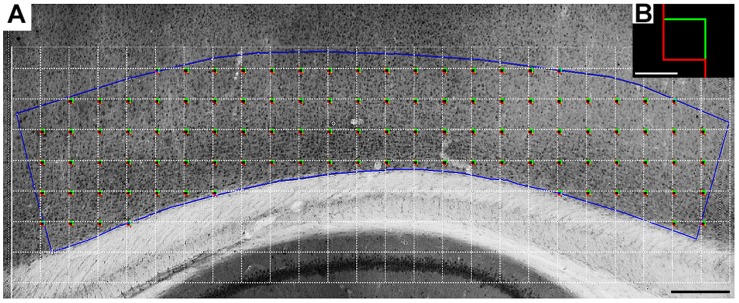
A representative low-power photomicrograph of a rat Egr-1-immunolabeled section of the somatosensorial infragranular cortical layers. **(A)** Blue line contour shows the border of the region of interest in a sagittal section. The white grid indicates the intervals between the square red/green boxes (shown larger in panel **B**) and shows the systematic random sampling approach. The total number of counting boxes in each section was proportional to the area covered by the somatosensory cortex infragranular layers. The antero-posterior limits of the contour were drawn using lines orthogonal to the pia mater. These lines originated at the border of the underlying hippocampal pyramidal cell layer. Because the area and the volume inside the contours could change within and between experimental groups, we estimated and compared volumetric cell densities rather than the total number of cells. Scale bar, 300 μm **(A)**, 20 μm **(B)**.

The histological section contours were digitized directly from the sections using a low-power 4 × objective on a Nikon (Eclipse 80i) microscope equipped with a motorized stage (MAC5000, Ludl Electronic Products, Hawthorne, NY, United States). This system was coupled to a computer running Stereo Investigator software (MBF Bioscience, Williston, VT, United States) that was used to store and analyze the *x*, *y*, and *z* coordinates of the digitized points. To identify and to unambiguously count the objects of interest in the dissector probe, the low-power objective was replaced with a Plan Fluorite 100 × objective (Nikon, NA 1.3, DF = 0.19 μm) (Gomes et al., 2019) to count Egr-1 cells (Figure 2).

The stereological estimations began with delimitation of the region of interest in sagittal sections (Carvalho-Paulo et al., 2017; Gomes et al., 2019). We projected orthogonal lines toward the pia mater of the somatosensory cortex starting from the tips of the hippocampal pyramidal cell layer (Figure 2). We also defined a horizontal line on the inferior margin of layer IV and another at the bottom of layer VI. Within this rectangular area, we counted the immunolabeled cells inside the counting boxes. As detailed by Carvalho-Paulo et al. (2017) (Carvalho-Paulo et al., 2017), the thickness of the section was carefully assessed at each counting site using a high-magnification objective, and the microscope’s fine focus was used to determine the immediate defocus above (top of section) and below (bottom of section). As noted previously, because this rectangular contour in the sections can vary from section to section in each animal, we estimated the cell density (cells/μm^3^). For this purpose, we estimated the total number of objects of interest using the volume of the rectangular area of interest. Thus, all counts were normalized using the planimetric volume estimates of the Stereoinvestigator^®^.

All sampled objects that were put into focus inside each counting frame were counted and added to the total marker sample, on condition that they were entirely within the counting frame or intersected the acceptance lines without touching the rejection lines (Gundersen and Jensen, 1987; Gomes et al., 2019). Supplementary Tables S1–S13 show the experimental parameters from the optical fractionator for the Egr-1–immunolabeled cell counts. The grid sizes were adapted to achieve an acceptable coefficient of error (CE). The experimental parameters for cell counting were established previously and were applied uniformly to all animals studied.

The cell number determination used for the cell density calculation in the “optical fractionator method was based on a random and systematic distribution of counting blocks in a series of sections containing the region of interest, all with the same probability of being sampled. The optical fractionator determined the number of cells by multiplying the number of objects inside each counting box by the values of three ratios: (i) the ratio between the number of sections sampled and the total number of sections (section sampling fraction, ssf); (ii) the ratio of the counting box and the area of the grid (area sampling fraction, asf); and (iii) the ratio between the height of the counting frame and the section thickness after histological processing (thickness sampling fraction, tsf). The total number of cells was obtained by the following equation” as earlier described (Carvalho-Paulo et al., 2017; Gomes et al., 2019):

N=Σ⁢Q×1/ssf×1/asf×1/tsf

Here, *N* is the total number of cells, and Σ*Q* is the number of counted objects (West et al., 1991).

The cell density was calculated as follows:

Celldensity(numberofcells/μm)3=N/measuredvolume

Data from the groups of animals were tested for statistical normality, and a few outliers were eliminated from the data set. Results from the optical fractionator were analyzed using two-way ANOVA to compare time recording-matched groups (2 h group and 6 h group) with different numbers of CSD episodes (high number versus low number) and hemispheres (all pairwise multiple comparison procedures, Holm–Sidak method). Pairwise multiple comparisons with statistically significant differences were checked using *t*-tests (with samples with equal and unequal variances). The significance level for statistical differences was set at alpha <0.05 (i.e., at a 95% confidence level). Statistical analyses were performed using Excel for Windows^®^, and GraphPad Prisma 6.

## Results

**FIGURE 3:**
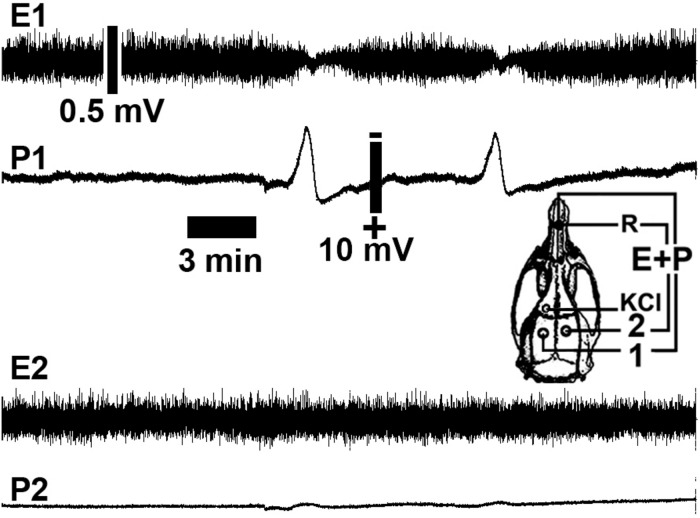
Representative cortical spreading depression (CSD) and electrocorticogram recordings from a rat in a 6-h recording group showing a low number of CSD episodes (two CSD episodes). The spontaneous electrical activity (electrocorticogram; E) and the slow change in potential (P) of CSD were recorded at two points (point 1 in the left hemisphere and point 2 in the right hemisphere) on the rat cortical surface. The skull diagram shows recording points 1 and 2, the point on the ipsilateral hemisphere that was stimulated with KCl to elicit CSD (KCl), and the point over the nasal bones where the common reference electrode (R) was placed. Note that CSD was restricted to the stimulated (left) hemisphere. Calibration bars show the respective values.

As described previously (Abadie-Guedes et al., 2008), CSD was consistently induced by 2% KCl applied to a cortical point in the frontal region. By the time that CSD was elicited, it could be recorded using the two other electrodes on the left side of the parietal cortex, the stimulated hemisphere. Figure 3 shows representative electrophysiological recordings [As stereotaxic coordinates, it was applied: stimulation hole with KCl, AP = +1; ML = 1; recording position 1(ipsilateral hemisphere): AP = –3; ML = 2; recording position 2 (contralateral hemisphere): AP = – 3; ML = 2].

Table 2 shows the density of Egr-1–positive cells in the experimental groups. The coefficients of biological variation were estimated using CVB^2^ = CV^2^ – CE^2^ (where CE is the coefficient of error; CV is the coefficient of variation; and CVB is the coefficient of biological variation). Values were expressed as a percentage of the coefficient of variation. The coefficient of error was considered adequate when it contributed less than the coefficient of biological variation to the overall coefficient of variation (Slomianka and West, 2005; Guerreiro-Diniz et al., 2010). All stereological parameters used to count Egr-1–positive cells using optical fractionator method are shown in Supplementary Tables S1–S13.

**TABLE 2:** Stereological determination of Egr-1–positive cell density on the left and right hemispheres at the infragranular layers of the somatosensorial cortex for 2 and 6 h of recording of the induction of cortical spreading depression (CSD) in adult rats.

**Group**	**Animal**	**Egr-1 cell**	**SCE**	**Thickness**	**Egr-1 cell**	**SCE**	**Thickness**
		**density RH**	**RH**	**RH**	**density LH**	**LH**	** LH**
2 h recording High number of CSD episodes	4	77,075.12	0.04	28.30	163,850.09	0.03	29.16
	6	66,525.43	0.04	29.57	104,606.94	0.03	30.16
	14	67,778.58	0.04	27.92	147,191.29	0.03	28.99
	24	122,969.38	0.03	28.61	110,417.15	0.03	29.72
	13	63,254.55	0.06	27.05	98,960.69	0.04	28.41
	15	75,121.43	0.05	24.15	109,653.23	0.04	26.97
	Mean	**78,787.53**	**0.04**	**27.60**	**122,446.563**	**0.03**	**28.90**
	SD	22,276.52	0.009	1.87	26,475.53	0.006	1.12
2 h recording Low number of CSD episodes	3	79,707.50	0.06	26.25	128,828.49	0.05	34.47
	11	63,274.85	0.05	25.06	105,694.41	0.05	25.45
	29	65,865.10	0.05	29.92	81,687.37	0.04	31.08
	31	78,962.42	0.04	31.23	97,698.16	0.04	29.79
	34	56,375.91	0.05	29.87	87,055.36	0.04	29.87
	Mean	**68,837.16**	**0.05**	**28.47**	**100,192.76**	**0.04**	**30.13**
	SD	10,194.86	0.005	2.66	18,517.14	0.006	3.23
6 h recording High number of CSD episodes	18	119,353.3238	0.04	31.08	79,166.98099	0.06	31.34
	20	159,225.4106	0.04	29.97	146,291.4382	0.04	31.08
	26	87,300.06771	0.05	29.58	103,398.8471	0.04	30.30
	27	118,105.1301	0.03	28.67	128,295.7078	0.03	28.14
	28	127,835.8445	0.04	30.51	119,502.0794	0.04	29.42
	Mean	**122,363.9553**	**0.04**	**29.96**	**115,331.0107**	**0.04**	**30.05**
	SD	25,716.66	0.006	0.92	79,166.98099	0.010	1.31
6 h recording Low number of CSD episodes	29 rep	79,578.4467	0.03	30.55	77,361.2809	0.04	29.78
	30	78,495.4937	0.04	30.44	82,902.15573	0.04	29.22
	32	78,738.9635	0.04	29.75	92,226.46693	0.04	30.37
	33	55,357.8865	0.05	29.09	69,011.61588	0.04	30.58
	39	73,257.0813	0.04	28.65	59,969.65573	0.05	28.03
	Mean	**73,085.5743**	**0.04**	**29.69**	**76,294.24**	**0.04**	**29.60**
	SD	10,218.68	0.007	0.83	12,431.4887	0.004	1.023
2 h recording Sham	9	72,696.24085	0.04	29.67	72,243.4081	0.04	27.29
	10	72,077.6669	0.04	26.75	66,980.1333	0.04	28.15
	19	69,493.47595	0.04	30.02	53,485.9831	0.05	29.17
	Mean	**71,422.46124**	**0.04**	**28.81**	**64,236.5081**	**0.04**	**28.20**
	SD	8,698.9398	0.0015	1.80	9,675.012	0.003	0.94
6 h recording Sham	36	59,427.30555	0.04	27.29	89,995.3672	28.49	0.04
	37	57,573.96491	0.04	28.15	73,798.953	29.19	0.04
	38	46,997.21448	0.05	29.17	51,662.4134	29.55	0.04
	Mean	**54,666.16165**	**0.04**	**28.20**	**71,8189112**	**29.08**	**0.04**
	SD	6,705.84	0.003	0.94	19,243.0315	0.54	0.005

*2 and 6 h; 2 and 6 h of recording; high and low number, high and low number of CSD episodes; sham, zero episodes; LH and RH, left and right hemispheres; SCE, Schaeffer coefficient error; SD, standard deviation.*

**FIGURE 4:**
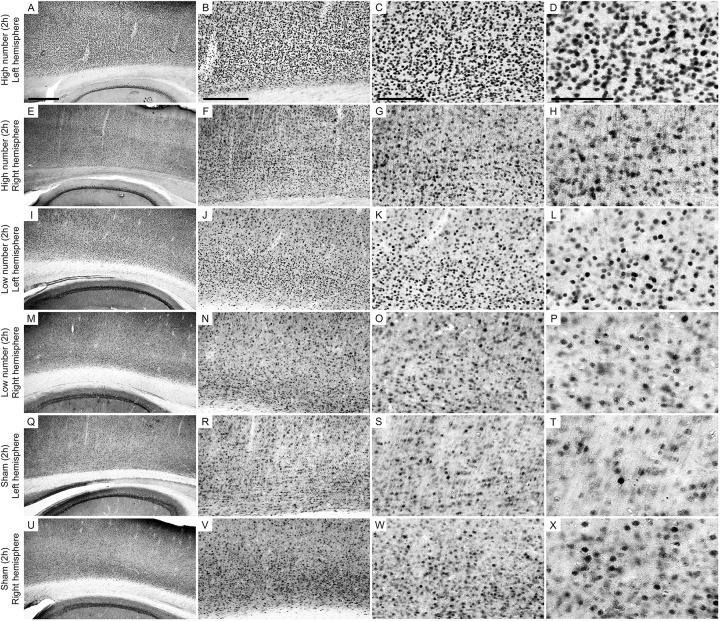
Low-power and high-power photomicrographs of the infragranular somatosensorial (ss) cortical layers in the indicated 2 h recording groups. The sections that were immunolabeled with anti-Egr-1 antibody showed qualitative differences between the 2 h recording groups: high number of CSD episodes in the left/ipsilateral hemisphere **(A–D)**; high number of CSD episodes in the right/contralateral hemisphere **(E–H)**; low number of CSD episodes in the left/ipsilateral hemisphere **(I–L)**; low number of CSD episodes in the right/contralateral hemisphere **(M–P)**; sham left/ipsilateral hemisphere **(Q–T)**; and sham right/contralateral hemisphere **(U–X)**. Scale bars in panels **(A,E,I,M,Q,U)** = 500 μm; in panels **(B,F,J,N,R,V)** = 300 μm; in panels **(C,G,K,O,S,W)** = 150 μm; and in panels **(D,H,L,P,T,X)** = 100 μm.

**FIGURE 5:**
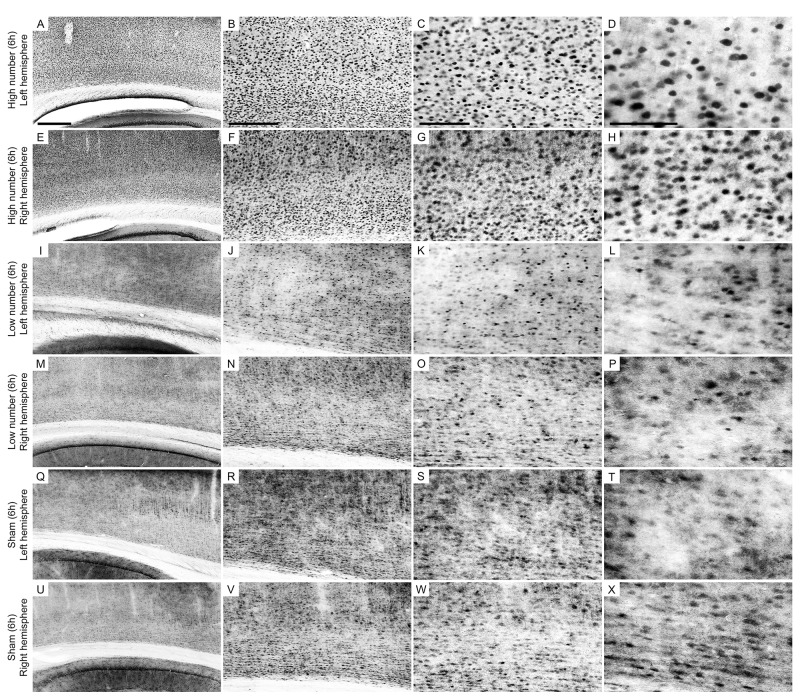
Low-power and high-power photomicrographs of the infragranular somatosensorial (ss) cortical layers in the indicated 6 h recording groups. The sections that were immunolabeled with anti-Egr-1 antibody showed qualitative differences between the 6 h recording groups: high number of CSD episodes in the left/ipsilateral hemisphere **(A–D)**; high number of CSD episodes in the right/contralateral hemisphere **(E–H)**; low number of CSD episodes in the left/ipsilateral hemisphere **(I–L)**; low number of CSD episodes in the right/contralateral hemisphere **(M–P)**; sham left/ipsilateral hemisphere **(Q–T)**; and sham right/contralateral hemisphere **(U–X)**. Scale bars in panels **(A,E,I,M,Q,U)** = 500 μm; in panels **(B,F,J,N,R,V)** = 300 μm; in panels **(C,G,K,O,S,W)** = 150 μm; and in panels **(D,H,L,P,T,X)** = 100 μm.

Figures 4, 5 show photomicrographs from an area of interest that illustrate the qualitative differences in cells immunolabeled with anti-Egr-1 antibody in the 2 and 6 h recording groups. The images were selected from individuals that had cell densities that were close to the average values of their groups.

**FIGURE 6:**
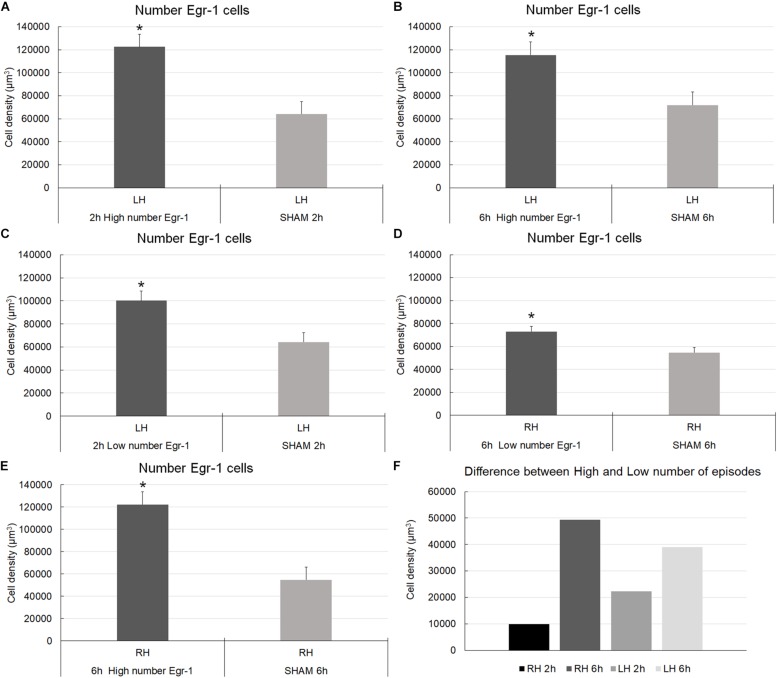
Graphic representation of the density of Egr-1–positive cells in the somatosensorial cortex. The graphs show stereological estimates of Egr-1–positive cell density (μm^3^; mean) in the infragranular layers of the somatosensorial cortex according to the indicated recording time (2 or 6 h) and according to the number of episodes of cortical spreading depression (high number and low number). **(A–E)** (^∗^) Statistically significant differences (parametric statistical analyses using *t*-tests – with samples with equal and unequal variances); **(F)** Differences in mean of stereological numerical estimates between the groups (higher number of CSD episodes minus who showed smaller number of episodes); overall significance level, 0.05; 2 and 6 h, 2 and 6 h of recording; high and low number, high and low number of CSD episodes; sham, zero episodes.

The cell density analysis (number of cells/μm^3^) showed that for the left/ipsilateral hemisphere, the number of CSD episodes (comparison between high and low number of episodes groups) was significant for the long-term register (6 h) [*F*(1,17) = 10.30; *p* = 0.0051] (Figure 6). However, the recording time and the interaction between them were not significant [*F*(1,17) = 2.637; *p* = 0.1228; *F*(1,17) = 0.7731; *p* = 0.3918, respectively] (Figure 6). Concerning about right/contralateral hemisphere, the number of CSD episodes, the recording times, and the interaction between them were significant [*F*(1,16) = 12.10; *p* = 0.0031; *F*(1,16) = 7.462; *p* = 0.0148; *F*(1,16) = 5.012; *p* = 0.0397, respectively]. Thus, in the right/contralateral hemisphere, despite the non-direct induction of CSD, the Egr-1**–**positive cells density were identified in a manner that was similar to that in the hemisphere in which CSD was induced, the left/ipsilateral one (Figure 6). We compared those with a higher number of CSD episodes less those that showed smaller number of episodes. We obtained a positive results showing that the estimates were higher in the groups that exhibited the largest number of CSD episodes. Thus, a correlation between cell density and number of episodes exists. The difference was also greater in those animals that remained longer time in electrophysiological record (6 h), that was independent of hemisphere.

**FIGURE 7:**
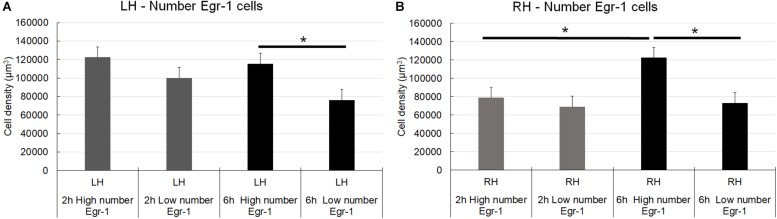
Graphic representation of the density of Egr-1–positive cells in the somatosensorial cortex. The graphs show stereological estimates of Egr-1–positive cell density (μm^3^; mean) in the infragranular layers of the somatosensorial cortex according to the indicated recording time (2 or 6 h) and according to the number of episodes of cortical spreading depression (high number and low number). **(A)** Left hemisphere; **(B)** Right hemisphere. Horizontal bars and (^∗^) indicate statistically indicate statistically significant differences using two-way analysis of variance, Holm-Sidak method; overall significance level, 0.05; 2 and 6 h, 2 and 6 h of recording; high and low number, high and low number of CSD episodes; sham, zero episodes.

Pairwise multiple comparisons showed statistically significant differences in Egr-1–positive cell density related to the 6 h recording groups as follows: 6 h high number LH group (115,331.01 ± 11,389.12) versus 6 h low number LH group (76,294.23 ± 5,559.53), *p* < 0.01; and 6 h high number RH group (122,363.95 ± 11,500.83) versus 6 h low number RH group (73,085.57 ± 4,569.93), *p* < 0.05 (mean ± S.E.) (Figure 6). It was possible that the number of CSD episodes and the long-term recording influenced Egr-1 expression. Indeed, we found differences in Egr-1–positive cell density as follows: 6 h high number RH group (122,363.95 ± 11,389.12) compared to 2 h high number RH group (78,787.52 ± 9,094.35), *p* < 0.05, now related to right/contralateral hemisphere (Figure 7).

There were statistically significant differences in Egr-1–positive cell density in the left/ipsilateral hemispheres in the 2 h recording groups (in both the high and low number of episodes groups) relate to the sham 2 h LH group: 2 h high number LH group (122,446.56 ± 10,808.59) *versus* sham 2 h LH group (64,236.50 ± 5,585.87) (*p* < 0.005) and, 2 h low number LH group (100,192.75 ± 8,281.11) *versus* sham 2 h LH group (64,236.50 ± 5,585.87) (*p* < 0.05). However, related to 6 h of recording, concerning to the high number of episode group, difference was detect at both hemisphere, left/ipsilateral and right/contralateral when they were compared to respective sham groups: 6 h high number of episodes LH group showed significantly higher cell density than the sham 6 h LH group (115,331.01 ± 11,389.12 versus 71,818.91 ± 11109.96, respectively; *p* < 0.05), and by the same way, the comparison between 6 h high number RH (122,363.95 ± 11,500.83) *versus* sham 6 h RH (54,666.16 ± 4,569.930; *p* < 0.05), and 6 h low number RH (73085,57 ± 8,281.11) *versus* sham 6 h RH (54,666.16 ± 4,569.930; *p* < 0.05) (Figure 7).

**FIGURE 8:**
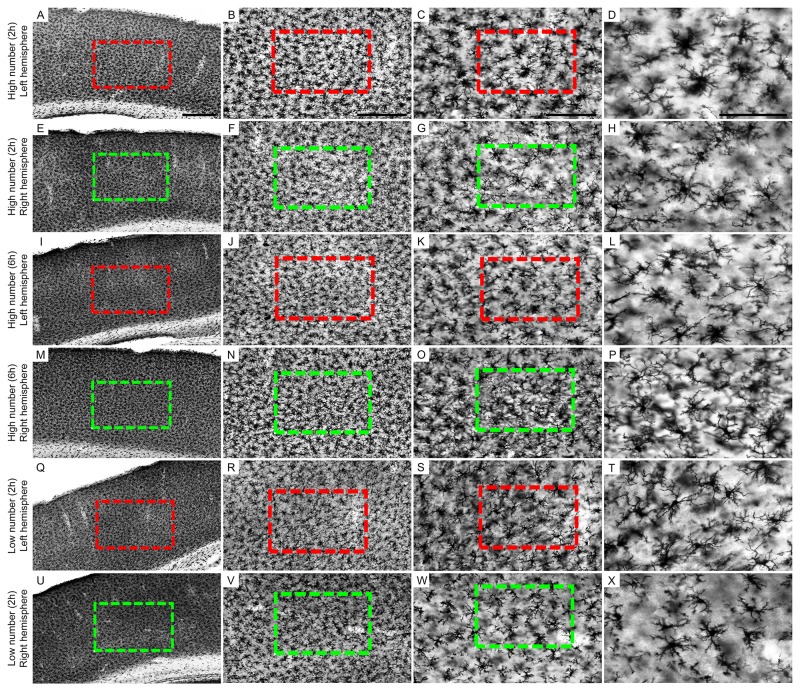
Low-power and high-power photomicrographs of the somatosensorial (ss) cortical layers in the indicated 6 and 2 h recording groups. The sections that were immunolabeled with anti-Iba-1 antibody showed qualitative differences between the 6 and 2 h recording groups: high number of CSD episodes (2-h recording) in the left/ipsilateral hemisphere **(A–D)**; high number of CSD episodes (2-h recording) in the right/contralateral hemisphere **(E–H)**; high number of CSD episodes (6-h recording) in the left/ipsilateral hemisphere **(I–L);** high number of CSD episodes (6-h recording) in the right/contralateral hemisphere **(M-P);** low number of CSD episodes (2-h recording) in the left/ipsilateral hemisphere **(Q–T)**; low number of CSD episodes (2-h recording) in the right/contralateral hemisphere **(U–X)**. Scale bars in panels **(A,E,I,M,Q,U)** = 500 μm; in panels **(B,F,J,N,R,V)** = 300 μm; in panels **(C,G,K,O,S,W)** = 150 μm; and in panels **(D,H,L,P,T,X)** = 100 μm.

We also qualitatively tested immunostaining for microglia (anti-Iba 1) in groups where a higher density of Egr-1 positive cells was identified [High number (2 h) ipsilateral, High number (6 h) ipsilateral; and High number (6 h) contrateral hemisphere)], adding groups where the density did not reach the same levels [Low number (2 h) ipsilateral; (2 h) contralateral and High number (2 h) contrateral hemisphere], and we were able to identify also in the group High number (6 h) contrateral hemisphere the suggestion of contralateral microglial morphological changes (Figure 8).

## Discussion

This was the first study using unbiased stereological methods to quantify the number of Egr-1–positive cells in response to CSD in the infragranular layers of the somatosensory cortex. We quantified the expression of Egr-1 cells calculating their density and we identified its remarkable presence with 2 h of recording, high number of CSD episodes and in the elicited hemisphere; this expression remaining high even with 6 h of recording and becoming significant also in these conditions, for the right hemisphere.

We also present a qualitative panel involving immunolabeling for microglia, which suggested microglial morphological changes in the right/contralateral hemisphere. Further experiments demonstrating consequences of expression extension and presence of Egr-1 positive cells should still contribute to the findings presented here.

Little is known about the of Egr-1 expression in a cytoarchitecture-dependent fashion in the cortex but close relationship of this area to the self-propagating wave of depolarization on the somatosensory cortex was focused at the present work. What we identified was that even in the suppression of high number of episodes, the expression of Egr-1 remains high and with contralateral reflexes. We reinforce the need to study other areas and with complementary methodology to observe the effects of Egr-1 expression. Fujita et al. (2016) identified that the velocity of SD propagation in the neocortex, related to the somatosensory, motor, and granular insular cortices, was higher than that in the paleocortex, suggesting “that cortical cytoarchitectonic features, which possibly involve the distribution of astrocytes, are crucial for regulating the velocity of SD propagation in the cerebral cortex.”

Migraine is described as a chronic primary brain disorder with episodic clinical manifestation and patients with migraine with aura often experience a variety of visual and somatosensory phenomena and disturbances of higher cortical functions (Petrusic and Zidverc-Trajkovic, 2014). Analysis of different cortical regions in CSD may provide important information about their involvement in these phenomena. The somatosensory cortex is related as a region undergoing a wave of depolarization and thus, neurological disorders of the migraine aura (Hougaard et al., 2017), and the effects of these cortical events on intrinsic cerebral connectivity during migraine aura attacks have also been poorly explored. Hougaard et al. (2017) identified a marked increase in connectivity during attacks between the left pons and the left primary somatosensory cortex including the head and face somatotopic areas corresponding well with the majority of patients reporting right-sided pain.

Although earlier studies reported changes to Egr-1 with CSD (McKee et al., 2006), none evaluated this region to investigate the potential influences of time and number of CSD episodes on Egr-1 expression. The IEGs are a single group of genes that are expressed as part of the first transcriptional wave after neuronal activation (Sommerlandt et al., 2018). IEGs broadly encode transcription factors that help maintain cellular homeostasis and neuronal plasticity. In the present study, we found that a higher number of CSD episodes resulted in higher Egr-1–positive cell density in the infragranular layers of the somatosensorial cortex in the 2- and 6-h post-induction recordings. The 6 h recording groups had a higher estimated density of Egr-1–positive cells in both hemispheres, suggesting a possible contralateral answer.

### Recordings Over Time and Over Multiple CSD Episodes Showed That the Contralateral Somatosensorial Cortex Overexpressed Egr-1–Positive-Cells

Repeated SDs spread not only concentrically from the ischemic zone but also cycled around the center if there was a permanently depolarized core (Woitzik et al., 2013; Dreier et al., 2017). In accordance with this, we detected a high density of Egr-1–positive cells in the contralateral (right) hemisphere in the 6 h recording group with a high number of CSD waves. Because IEGs can be used as molecular markers of neural connections, as suggested by Sommerlandt et al. (2018), we speculated that callosal connections might explain the contralateral Egr-1 expression that we observed (Mun-Bryce et al., 2006; Martins, 2007; Pinto and Guedes, 2008). It is important to recognize that repeated SDs may have longer-lasting consequences i.e., consequences for more than 6 h after CSD episodes. Indeed, recurrent SDs cause reactive astrocytosis, as evidenced by dramatic glial fibrillary acidic (GFAP) protein staining and hypertrophy that persists for several weeks (Kraig et al., 1991). As described by Honkaniemi et al., 1997) (Honkaniemi et al., 1997), prolonged expression of the fos/jun IEGs correlates with slowly dying neurons after cerebral ischemia. Activation, along with hippocampal and thalamic IEG induction efferent cortical pathways to these regions, could induce these IEGs in the cortex.

Multiple CSD events, but not single CSD events, significantly increase neuropeptide calcitonin gene-related peptide (CGRP) mRNA levels 24 h post-CSD (Wang et al., 2016). This is limited to the ipsilateral rat cerebral cortex; an increase in CGRP is detected in the ipsilateral frontal, motor, somatosensory, and visual cortices but not in the contralateral cortex. Cortical TRPA1 (transient receptor potential ankyrin type-1) activation plays a critical role in regulating cortical susceptibility to CSD, and the channel signaling during CSD involves CGRP. Thus, these channels might be a potential target for preventing migraine aura (Jiang et al., 2018).

### Optical Fractionator Data Indicate a Higher Number of Estimated Egr-1–Immunolabeled Cells in the Contralateral Hemisphere

Fos expression is increased specifically in the magnocellular region of the hypothalamic paraventricular nucleus (PVN) and by the same way, in the ipsilateral cortex, whereas decreased Fos expression is noted in both the parvocellular region of the PVN and in the entire cortex contralateral to the CSD site (Iqbal Chowdhury et al., 2003). Consistent with reduced Fos expression, approximately 40% of neurons in the contralateral cortex show suppressed electrical activity during CSD, suggesting that the CSD can affect Fos expression in distinct areas. These results suggest that CSD may have a differential effect depending on the involved cerebral area with reflexes on the contralateral side, but previous studies only used a bidimensional counting method. We estimated the cell density of Egr-1–immunolabeled cells as a function of time and number of CSD episodes and according to the hemisphere in which CSD was elicited. This ensures that there was an adequate estimation of the total number of cells and cell density within the area of interest (Cruz-Orive, 1994; Schmitz, 1998; Schmitz and Hof, 2005).

In the present report, the acceptable level of errors related to the stereological estimations was defined by the ratio between the intrinsic error introduced by the methodology and the coefficient of the variation (Glaser and Wilson, 1998; Slomianka and West, 2005; Carvalho-Paulo et al., 2017). The CE indicates the accuracy of the estimated cell number. A CE value ≤0.05 was deemed appropriate for the present study because variance introduced by the estimation procedure contributes little to the observed group variance (Slomianka and West, 2005). The calculation of biological variation was defined as CVB^2^ = CV^2^ – CE^2^ (where CE is the coefficient of error, CV is the coefficient of variation, and CVB is the coefficient of biological variation) expressed as a percentage of the coefficient of variation. The coefficient of error is considered adequate when it contributes less than biological variation to the overall coefficient of variation, as in this study.

### The Estimated Egr-1–Positive Cell Density Was Influenced by the Recurrent CSD Episodes

Single-photon emission computed tomography (SPECT) data indicates that CSD clustering may occur in hemiplegic migraine, a migraine subtype that is characterized by prolonged aura with motor weakness (Iizuka et al., 2012, 2015; Takizawa et al., 2019). Nevertheless, the pathophysiological differences between single and clustering SD remain unclear. Takizawa et al. (2016, 2017, 2019) recently reported that the release of the high-mobility group box 1 (HMGB1) protein from cortical neurons increases with increasing CSD induction in a rodent model. Therefore, CSD clusters evoke far greater HMGB1 release than do single events. In accordance with this, we observed an increase in Egr-1*–*positive cells as a function of time and number of CSD events. Although HMGB1 was originally identified as a non-histone nuclear protein, it may acts as a cytokine when released into the extracellular space (Harris et al., 2012; Takizawa et al., 2019). However, the pathophysiological events downstream of multiple episodes of CSD-induced HMGB1 release remain to be explored. For example, after single or multiple CSD episodes, no significant differences are detected in the bi-dimensional density of cortical Iba1-positive microglia, and the microglia do not show altered expression of major histocompatibility antigen type II, a marker of immune activation (Takizawa et al., 2017). Instead, multiple CSDs increase the microglial expression of cathepsin D, a major lysosomal acid hydrolase, maybe identifying a molecular control mechanism for microglial response and the subsequent responses induced by clustering of SDs/depolarizations associated with stroke and hemiplegic migraine (Takizawa et al., 2017, 2019).

### Possible Interpretations and Conclusion

Cortical spreading depressions may be the largest pathophysiological disruptor of cerebral gray matter homeostasis and could be an important mechanism underlying tissue damage (Dreier et al., 2013). Understanding the involvement of Egr-1 in epidemiologically relevant diseases such as migraine and their relationship to CSD may be important for the development of new therapeutic strategies. We found that in an acute interval after CSD induction, i.e., 2 h, Egr-1 expression increased significantly in the hemisphere in which CSD was induced. Further, its expression was maintained over a 6-h period when a high number of CSD episodes were induced. Although the underlying mechanism remains to be elucidated, the observation over a long recording interval showed that a high Egr-1 density cells also occurs contralaterally, suggesting that unknown molecular signaling changes associated with CSD may be transmitted from one hemisphere to the other.

## Data Availability Statement

All datasets generated for this study are included in the manuscript/Supplementary Files.

## Ethics Statement

The animal study was reviewed and approved by Ethics Committee for Animal Research of the Federal University of Pernambuco, Brazil (protocol approval no. 0037/2016).

## Author Contributions

MS, DD, JB, CD, and RG participated in the development and methodological design, collection and treatment of data, and analysis and interpretation of data and writing. JR, RA-G, FSM, and TF participated in the collection and processing of data. DA participated in the development and methodological design, supervision, analysis and interpretation of data and writing.

## Conflict of Interest

The authors declare that the research was conducted in the absence of any commercial or financial relationships that could be construed as a potential conflict of interest.
