# Supplementary material for: Stereological Analysis of Early Gene Expression Using Egr-1 Immunolabeling After Spreading Depression in the Rat Somatosensory Cortex
Source: Front Neurosci. 2019 Sep 25;13:1020. doi: 10.3389/fnins.2019.01020 (PMC6774394; doi:10.3389/fnins.2019.01020)
Supplement: Supplementary file 1 [file Table_1.docx]

**S1 Table Stereological parameters for the Egr-1 positive cells in the somatosensorial cortex of adult rats.** N = total number of objects of interest counted, SSF = Section Sampling Fraction, ASF = Area, SSC = Somatosensorial Cortex.

| Group | Animal | a(frame)  (µm) | A(x,y step)  μm^2^ | ASF | TSF | SSF | N° Counting  Frames | N° Sections  SSC | N |
| --- | --- | --- | --- | --- | --- | --- | --- | --- | --- |
| 2h recording High number of CSD episodes  RH | 4 | 20X20 | 150X150 | 0.0178 | 0.35 | 1/3 | 325 | 5 | 163439 |
|  | 6 | 20X20 | 150X150 | 0.0178 | 0.34 | 1/3 | 432 | 6 | 184144 |
|  | 14 | 20X20 | 150X150 | 0.0178 | 0.36 | 1/3 | 405 | 5 | 180168 |
|  | 24 | 20X20 | 150X150 | 0.0178 | 0.35 | 1/3 | 363 | 5 | 282239 |
|  | 13 | 20X20 | 150X150 | 0.0178 | 0.38 | 1/3 | 280 | 5 | 116693 |
|  | 15 | 20X20 | 150X150 | 0.0178 | 0.42 | 1/3 | 294 | 5 | 145933 |

**S2 Table Stereological parameters for the Egr-1 positive cells in the somatosensorial cortex of adult rats.** N = total number of objects of interest counted, SSF = Section Sampling Fraction, ASF = Area, SSC = Somatosensorial Cortex.

| Group | Animal | a(frame)  (µm) | A(x,y step)  μm^2^ | ASF | TSF | SSF | N° Counting  Frames | N° Sections  SSC | N |
| --- | --- | --- | --- | --- | --- | --- | --- | --- | --- |
| 2h recording High number of CSD episodes  LH | 4 | 20X20 | 150X150 | 0.0178 | 0.34 | 1/3 | 346 | 5 | 367183 |
|  | 6 | 20X20 | 150X150 | 0.0178 | 0.33 | 1/3 | 542 | 6 | 360509 |
|  | 14 | 20X20 | 150X150 | 0.0178 | 0.35 | 1/3 | 283 | 5 | 265424 |
|  | 24 | 20X20 | 150X150 | 0.0178 | 0.34 | 1/3 | 368 | 5 | 261841 |
|  | 13 | 20X20 | 150X150 | 0.0178 | 0.36 | 1/3 | 376 | 6 | 238340 |
|  | 15 | 20X20 | 150X150 | 0.0178 | 0.38 | 1/3 | 345 | 6 | 241488 |

**S3 Table Stereological parameters for the Egr-1 positive cells in the somatosensorial cortex of adult rats.** N = total number of objects of interest counted, SSF = Section Sampling Fraction, ASF = Area, SSC = Somatosensorial Cortex.

| Group | Animal | a(frame)  (µm) | A(x,y step)  μm^2^ | ASF | TSF | SSF | N° Counting  Frames | N° Sections  SSC | N |
| --- | --- | --- | --- | --- | --- | --- | --- | --- | --- |
| 2h recording Low number of CSD episodes  RH | 3 | 20X20 | 150X150 | 0,0178 | 0.39 | 1/3 | 235 | 5 | 120391 |
|  | 11 | 20X20 | 150X150 | 0,0178 | 0.41 | 1/3 | 225 | 5 | 91802 |
|  | 29 | 20X20 | 150X150 | 0,0178 | 0.33 | 1/3 | 426 | 5 | 181952 |
|  | 31 | 20X20 | 150X150 | 0,0178 | 0.31 | 1/3 | 313 | 5 | 161036 |
|  | 34 | 20X20 | 150X150 | 0,0178 | 0.33 | 1/3 | 501 | 6 | 182471 |

**S4 Table Stereological parameters for the Egr-1 positive cells in the somatosensorial cortex of adult rats.** N = total number of objects of interest counted, SSF = Section Sampling Fraction, ASF = Area, SSC = Somatosensorial Cortex.

| Group | Animal | a(frame)  (µm) | A(x,y step)  μm^2^ | ASF | TSF | SSF | N° Counting  Frames | N° Sections  SSC | N |
| --- | --- | --- | --- | --- | --- | --- | --- | --- | --- |
| 2h recording Low number of CSD episodes  LH | 3 | 20X20 | 150X150 | 0.0178 | 0.29 | 1/3 | 294 | 6 | 240349 |
|  | 11 | 20X20 | 150X150 | 0.0178 | 0.40 | 1/3 | 237 | 5 | 156039 |
|  | 29 | 20X20 | 150X150 | 0.0178 | 0.33 | 1/3 | 504 | 6 | 261777 |
|  | 31 | 20X20 | 150X150 | 0.0178 | 0.34 | 1/3 | 337 | 5 | 210728 |
|  | 34 | 20X20 | 150X150 | 0.0178 | 0.34 | 1/3 | 446 | 7 | 248892 |

**S5 Table Stereological parameters for the Egr-1 positive cells in the somatosensorial cortex of adult rats.** N = total number of objects of interest counted, SSF = Section Sampling Fraction, ASF = Area, SSC = Somatosensorial Cortex.

| Group | Animal | a(frame)  (µm) | A(x,y step)  μm^2^ | ASF | TSF | SSF | N° Counting  Frames | N° Sections  SSC | N |
| --- | --- | --- | --- | --- | --- | --- | --- | --- | --- |
| 6h recording High number of CSD episodes  RH | 18 | 20X20 | 150X150 | 0.0178 | 0.32 | 1/3 | 347 | 6 | 265476 |
|  | 20 | 20X20 | 150X150 | 0.0178 | 0.33 | 1/3 | 304 | 5 | 310369 |
|  | 26 | 20X20 | 150X150 | 0.0178 | 0.34 | 1/3 | 297 | 5 | 169193 |
|  | 27 | 20X20 | 150X150 | 0.0178 | 0.35 | 1/3 | 456 | 6 | 342254 |
|  | 28 | 20X20 | 150X150 | 0.0178 | 0.33 | 1/3 | 327 | 5 | 269628 |

**S6 Table Stereological parameters for the Egr-1 positive cells in the somatosensorial cortex of adult rats.** N = total number of objects of interest counted, SSF = Section Sampling Fraction, ASF = Area, SSC = Somatosensorial Cortex.

| Group | Animal | a(frame)  (µm) | A(x,y step)  μm^2^ | ASF | TSF | SSF | N° Counting  Frames | N° Sections  SSC | N |
| --- | --- | --- | --- | --- | --- | --- | --- | --- | --- |
| 6h recording High number of CSD episodes  LH | 18 | 20X20 | 150X150 | 0.0178 | 0.32 | 1/3 | 319 | 5 | 163663 |
|  | 20 | 20X20 | 150X150 | 0.0178 | 0.32 | 1/3 | 326 | 5 | 307213 |
|  | 26 | 20X20 | 150X150 | 0.0178 | 0.33 | 1/3 | 337 | 6 | 225811 |
|  | 27 | 20X20 | 150X150 | 0.0178 | 0.36 | 1/3 | 478 | 6 | 400310 |
|  | 28 | 20X20 | 150X150 | 0.0178 | 0.34 | 1/3 | 361 | 5 | 279531 |

**S7 Table Stereological parameters for the Egr-1 positive cells in the somatosensorial cortex of adult rats.** N = total number of objects of interest counted, SSF = Section Sampling Fraction, ASF = Area, SSC = Somatosensorial Cortex.

| Group | Animal | a(frame)  (µm) | A(x,y step)  μm^2^ | ASF | TSF | SSF | N° Counting  Frames | N° Sections  SSC | N |
| --- | --- | --- | --- | --- | --- | --- | --- | --- | --- |
| 6h recording Low number of CSD episodes  RH | 29 REP | 20X20 | 150X150 | 0.0178 | 0.33 | 1/3 | 462 | 5 | 238682 |
|  | 30 | 20X20 | 150X150 | 0.0178 | 0.33 | 1/3 | 370 | 5 | 187246 |
|  | 32 | 20X20 | 150X150 | 0.0178 | 0.34 | 1/3 | 454 | 6 | 222297 |
|  | 33 | 20X20 | 150X150 | 0.0178 | 0.35 | 1/3 | 328 | 5 | 119346 |
|  | 39 | 20X20 | 150X150 | 0.0178 | 0.35 | 1/3 | 383 | 5 | 178369 |

**S8 Table Stereological parameters for the Egr-1 positive cells in the somatosensorial cortex of adult rats.** N = total number of objects of interest counted, SSF = Section Sampling Fraction, ASF = Area, SSC = Somatosensorial Cortex.

| Group | Animal | a(frame)  (µm) | A(x,y step)  μm^2^ | ASF | TSF | SSF | N° Counting  Frames | N° Sections  SSC | N |
| --- | --- | --- | --- | --- | --- | --- | --- | --- | --- |
| 6h recording Low number of CSD episodes  LH | 29 REP | 20X20 | 150X150 | 0.0178 | 0.34 | 1/3 | 359 | 5 | 175947 |
|  | 30 | 20X20 | 150X150 | 0.0178 | 0.35 | 1/3 | 256 | 5 | 138292 |
|  | 32 | 20X20 | 150X150 | 0.0178 | 0.33 | 1/3 | 337 | 5 | 197784 |
|  | 33 | 20X20 | 150X150 | 0.0178 | 0.33 | 1/3 | 390 | 5 | 171113 |
|  | 39 | 20X20 | 150X150 | 0.0178 | 0.36 | 1/3 | 371 | 5 | 144253 |

**S9 Table Stereological parameters for the Egr-1 positive cells in the somatosensorial cortex of adult rats.** N = total number of objects of interest counted, SSF = Section Sampling Fraction, ASF = Area, SSC = Somatosensorial Cortex.

| Group | Animal | a(frame)  (µm) | A(x,y step)  μm^2^ | ASF | TSF | SSF | N° Counting  Frames | N° Sections  SSC | N |
| --- | --- | --- | --- | --- | --- | --- | --- | --- | --- |
| 2h recording SHAM  RH | 9 | 20X20 | 150X150 | 0.0178 | 0.34 | 1/3 | 389 | 5 | 176461 |
|  | 10 | 20X20 | 150X150 | 0.0178 | 0.37 | 1/3 | 362 | 6 | 171876 |
|  | 19 | 20X20 | 150X150 | 0.0178 | 0.36 | 1/3 | 475 | 6 | 208075 |

**S10 Table Stereological parameters for the Egr-1 positive cells in the somatosensorial cortex of adult rats.** N = total number of objects of interest counted, SSF = Section Sampling Fraction, ASF = Area, SSC = Somatosensorial Cortex.

| Group | Animal | a(frame)  (µm) | A(x,y step)  μm^2^ | ASF | TSF | SSF | N° Counting  Frames | N° Sections  SSC | N |
| --- | --- | --- | --- | --- | --- | --- | --- | --- | --- |
| 2h recording SHAM  LH | 9 | 20X20 | 150X150 | 0.0178 | 0.36 | 1/3 | 331 | 5 | 151704 |
|  | 10 | 20X20 | 150X150 | 0.0178 | 0.33 | 1/3 | 287 | 5 | 123514 |
|  | 19 | 20X20 | 150X150 | 0.0178 | 0.33 | 1/3 | 475 | 5 | 161162 |

**S11 Table Stereological parameters for the Egr-1 positive cells in the somatosensorial cortex of adult rats.** N = total number of objects of interest counted, SSF = Section Sampling Fraction, ASF = Area, SSC = Somatosensorial Cortex.

| Group | Animal | a(frame)  (µm) | A(x,y step)  μm^2^ | ASF | TSF | SSF | N° Counting  Frames | N° Sections  SSC | N |
| --- | --- | --- | --- | --- | --- | --- | --- | --- | --- |
| 6h recording SHAM  RH | 36 | 20X20 | 150X150 | 0.0178 | 0.37 | 1/3 | 311 | 5 | 116262 |
|  | 37 | 20X20 | 150X150 | 0.0178 | 0.36 | 1/3 | 450 | 6 | 165809 |
|  | 38 | 20X20 | 150X150 | 0.0178 | 0.35 | 1/3 | 458 | 5 | 137199 |

**S12 Table Stereological parameters for the Egr-1 positive cells in the somatosensorial cortex of adult rats.** N = total number of objects of interest counted, SSF = Section Sampling Fraction, ASF = Area, SSC = Somatosensorial Cortex.

| Group | Animal | a(frame)  (µm) | A(x,y step)  μm^2^ | ASF | TSF | SSF | N° Counting  Frames | N° Sections  SSC | N |
| --- | --- | --- | --- | --- | --- | --- | --- | --- | --- |
| 6h recording SHAM  LH | 36 | 20X20 | 150X150 | 0.0178 | 0.35 | 1/3 | 313 | 5 | 176772 |
|  | 37 | 20X20 | 150X150 | 0.0178 | 0.35 | 1/3 | 373 | 6 | 176211 |
|  | 38 | 20X20 | 150X150 | 0.0178 | 0.34 | 1/3 | 411 | 5 | 134031 |

***S13 Table* Stereological determination of Egr-1–positive cell density.** Coefficient of biological variation on the left/ipsi and right/contralateral hemispheres at the infragranular layers of the somatosensorial cortex for 2 hours and 6 hours of recording of the induction of cortical spreading depression (CSD) in adult rats are determined at table.

CV= Coefficient of Variation; CE= Coefficient of Error; CBV= Coefficient of Biological Variation.

| Group | Mean (Egr-1 cell density) | SD | CV^2^ | CE^2^ | CBV^2^ | CBV ^2^/CV^2^ | CBV ^2^ (%CV^2^) |
| --- | --- | --- | --- | --- | --- | --- | --- |
| 2h recording High number of CSD episodes  RH | 78,787.53 | 22,276.52 | 0.07994288 | 0.00190067 | 0.07804221 | 0.97622466 | 97.6224657 |
| 2h recording High number of CSD episodes  LH | 122,446.563 | 26,475.53 | 0.046751564 | 0.001107281 | 0.045644283 | 0.976315635 | 97.63156355 |
| 2h recording Low number of CSD episodes  RH | 68,837.16 | 10194.86 | 0.02193392 | 0.00234568 | 0.01958825 | 0.89305707 | 89.305707 |
| 2h recording Low number of CSD episodes  LH | 100,192.76 | 18517.14 | 0.03415663 | 0.00182314 | 0.03233349 | 0.94662423 | 94.6624228 |
| 6h recording High number of CSD episodes  RH | 122,363.9553 | 25716.66 | 0.04416946 | 0.00153062 | 0.04263883 | 0.96534653 | 96.5346535 |
| 6h recording High number of CSD episodes  LH | 115,331.0107 | 79,166.98099 | 0.04875941 | 0.00165756 | 0.04710184 | 0.96600524 | 96.6005235 |
| 6h recording Low number of CSD episodes  RH | 73,085.5743 | 10,218.68 | 0.01954908 | 0.00165005 | 0.01789903 | 0.91559453 | 91.559453 |
| 6h recording Low number of CSD episodes  LH | 76,294.24 | 12,431.4887 | 0.0265499 | 0.00191963 | 0.02463027 | 0.92769721 | 92.7697212 |
| 2h recording  Sham  RH | 71,422.46124 | 8,698.9398 | 0.01483416 | 0.00175059 | 0.01308358 | 0.88198959 | 88.198959 |
| 2h recording  Sham  LH | 64,236.5081 | 9,675.012 | 0.02268502 | 0.00201341 | 0.02067161 | 0.9112451 | 91.1245104 |
| 6h recording  Sham  RH | 54,666.16165 | 6,705.84 | 0.01504767 | 0.00201341 | 0.01303426 | 0.86619807 | 86.6198071 |
| 6h recording  Sham  LH | 71,8189112 | 19,243.0315 | 0.07179089 | 0.00154444 | 0.07024645 | 0.978487 | 97.8486998 |

*2h and 6h; 2 hours and 6 hours of recording; high and low number, high and low number of CSD episodes; sham, zero episodes; LH and RH, left and right hemispheres; SCE, Schaeffer coefficient error; SD, standard deviation; CV, coefficient of variation; CE, coefficient of error; CBV, coefficient of biological variation.*
